# Supplementary material for: Are carbon emissions trading and green financial instruments synergistic? -Comprehensive quantitative research based on content analysis
Source: PLoS One. 2024 Mar 7;19(3):e0298601. doi: 10.1371/journal.pone.0298601 (PMC10919601; doi:10.1371/journal.pone.0298601)
Supplement: S1 Data — (ZIP) [file pone.0298601.s001.zip › Data/地级市Z_F工作报告.zip!/┴╔─■/║∙┬1⁄2╡║╩╨/└┤╫╘╣1⁄2╓┌║┼í╛┬φ┐╦╩2╛▌═°í┐_2018.docx]

各位代表：

　　现在，我代表市人民政府向大会报告工作，请予审议，并请市政协委员和列席会议的同志提出意见。

　　一、本届政府工作回顾

　　本届政府任期四年。

　　四年来，在市委的坚强领导下，在市人大、市政协的监督支持下，我们坚持稳中求进工作总基调，团结和带领全市人民，改革创新、攻坚克难，砥砺奋进、逆势而上，经济社会发展取得显著成绩。

　　四年来，我们主动适应经济发展新常态，加快振兴发展，实现了稳中有进、稳中向好。做实了经济数据，固定资产投资、财政收入快速增长，经济总量稳步提升，实现了位次前移。

　　四年来，我们深入推进供给侧结构性改革，协调发展三次产业，实现了效益增长、质量提升。农业经济更新换代、提质升级，工业经济筑底企稳、持续向好，传统服务业加快转型，现代服务业蓬勃发展。

　　四年来，我们全力实施项目牵动战略，狠抓项目建设，实现了结构调整、总量扩张。葫芦岛铝业、大唐热电等一批项目开工，渤船重工核电装备制造等一批项目投产，青山水库、建兴高速等一批基础设施项目建成，经济发展支撑能力显著增强。

　　四年来，我们着力破除体制机制障碍，深化各项改革，实现了全面推进、多点突破。深入推进国企改革，中冶葫芦岛有色混合所有制改革成为全省国企改革的样板。扎实推进公车制度、财税体制等各项改革，持续深化“放管服”改革、机构编制改革，营商环境明显改善。

　　四年来，我们积极融入“一带一路”建设，推进对外开放，实现了布局优化、实力增强。葫芦岛港实现外贸运输正常化。沿海经济带加快发展，园区基础设施完备，外资外贸双增，跨境电商、外贸海外仓、对外投资实现新突破。

　　四年来，我们大力推进生态文明建设，加强环境保护，实现了污染减少、环境改善。认真贯彻落实“大气十条”“水十条”“土十条”，实施“青山碧水蓝天净土”工程，持续开展污染防治，生态环境明显好转，人与自然更加和谐。

　　四年来，我们努力克服财政巨大困难，加大民生投入，实现了社会进步、民生改善。全面落实各项政策，文化教育事业不断发展，医疗卫生水平不断提高，社会保障体系基本形成，公共服务体系更加完善。举全市之力脱贫攻坚，贫困人口减少25.5万，脱贫攻坚取得阶段性成果。

　　二、2017年工作总结

　　各位代表，2017年是本届政府的收官之年。一年来，我们深入贯彻落实新发展理念和“四个着力”“三个推进”要求，把做大经济总量作为首要任务，抓住项目建设这个关键，狠抓作风转变，狠抓工作推进，狠抓任务落实，取得较好成绩。

　　预计2017年，地区生产总值700亿元，增长7%;规模以上工业增加值192亿元，增长10%;固定资产投资220亿元，增长23.6%;一般公共预算收入72.8亿元，增长19.9%;社会消费品零售总额500亿元，增长6%;城镇居民人均可支配收入27997元，增长6.3%;农村居民人均可支配收入11678元，增长6.3%。固定资产投资、规模以上工业增加值、一般公共预算收入增幅列全省第1位、总量实现位次前移，其它主要经济指标增幅均排全省前列。

　　(一)落实“五个围绕”，立足富民抓调整，立足长远抓产业，走上了摆脱雨养农业之路。发展设施农业，调减玉米面积30万亩，新增设施农业2万亩。发展品牌果业，建设精品科技示范园50个。发展海洋渔业，建成海洋牧场12万亩。发展认养农业、体验农业等新型业态，吸引城市资本向农村集聚，1.5万农民受益。发展农产品深加工，引进玉米、大豆、海产品深加工等重大项目，农产品加工园区新增企业28家。发展新型农业经营主体，建成市级示范合作社和家庭农场各30个。完善流通体系，建成农产品批发市场3个。夯实了现代农业基础。

　　(二)实施“六大工程”，做优存量，扩大增量，提高发展质量和效益，工业经济逐步摆脱困境。狠抓工业项目，推进扩能增效，实施亿元以上重点项目53个，竣工投产10个。延长产业链条，加强技术改造，培育经济增长点，新增规上工业企业30户。深化军民融合，打造五大板块，军民融合总产值超过120亿元，我市被确定为省级军民融合示范区。预计规上工业总产值680亿元，增长24%;实现利润20.6亿元，增长8%，工业实力显著提升。

　　(三)改造商贸物流等传统服务业，实施隆东国际城市综合体等重点项目23个，建设冷链物流重点项目4个。发展电商、会展等新兴业态，举办泳装博览会、避暑纳凉节、电竞大赛等大型节会，会展经济交易额增长11.5%;新建电商平台20个，电商交易额增长35%。我市被列入“国家电子商务示范市”创建城市。

　　落实“六个一工程”和“换脑工程”，推进旅游资源产品化，实施重点项目49个，竣工10个。创新服务模式，建成智慧旅游系统，开通旅游专列和观光巴士。旅游业总收入增长20%。我市被省政府批准为对接京津冀协同发展旅游先行区。

　　(四)落实“五个一批”，开展“项目年”活动，一批规模大、档次高、带动能力强、产业优势突出的重大项目顺利实施。200个亿元以上重点项目全部开复工，完成投资195亿元。葫芦岛铝业一期、建昌光伏发电等61个项目竣工投产。北方铜业电解铜、海洋核动力平台等重大项目即将达产，项目建设支撑作用凸显。

　　落实招商“三种方式”，压实主体责任，创新招商方式，转变招商方向，提高项目签约率、落地率。全年签约项目51个，引进省外内资131.6亿元，增长1.4倍。承接京津冀产业转移项目32个，开工建设18个，完成投资87亿元，招商引资成为工作常态。

　　(五)实施三城同盛，推进同城同质，全年落实重点项目75个。实施改造老城，启动道路畅通、住房改善、环境提升、设施完善、市民休闲、美化亮化6大工程，老城面貌得到改善。实施提升新城，开工建设万豪酒店、市民服务中心等重点项目，完成龙湾海滨综合治理、雨污分流工程，新城品质得到提升。实施连接兴城，开工建设欢乐龙湾、首山湖、泉海御龙湾等一批重点项目，实现规划同步、设施共建、公交互通，初步完成对接融合，拉开了城市发展格局。

　　完善城市路网，建设了城市外环路、海航路、天化路、海韵路、龙前街、龙绣街、海辰立交桥辅桥、海翔立交桥等14条道路，疏解了过境车辆，缓解了城市交通压力。夯实城市承载，4座城镇污水处理厂完成提标改造，市净水厂和水质检测中心竣工投入使用，让市民喝上了放心水。

　　(六)深化国资国企改革，完成央企“三供一业”分离移交，推进厂办大集体改革。市属企业集团规范运营，发挥了防风险、促融资等关键作用。深入推进“放管服”改革，调整行政职权事项43项，“双随机一公开”监管实现全覆盖。实施事业单位分类改革，清理收回事业编制5760个。启动16所公立医院改革，乡镇卫生院全部恢复公益性。落实“三去一降一补”任务，29家地方小煤矿全部关闭，去产能123万吨;房地产去库存178万平方米，增长1.5倍;减免企业各项税费超20亿元，改革引领经济发展的动力增强。

　　(七)主动融入“一带一路”建设，搭建开放平台，拓展开放载体，推动外向型经济加快发展。推进与营口港集团合作，加快港口建设和对外开放，葫芦岛港开通定期货运航线，实现首次外轮内运。兴城保税仓库获沈阳海关批准，滨海经济区成为辽宁自贸区协作区。以园区为载体，加快沿海经济带对外开放，引进德国钾盐二期、日本和杨德信环保等重点项目。拓展双向投资，新批外资项目9个，实际利用外资3885万美元;正业集团、七星钢管等企业实现对外投资。扩大外贸进出口，国际贸易“单一窗口”投入使用，外贸出口51亿元，增长53%，外贸进口50亿元，增长2.3倍，对外开放水平大幅提升。

　　(八)提升社会保障能力，推进就业创业，城镇登记失业率低于省控指标，零就业家庭保持动态为零，我市成为全国社保卡综合利用示范基地。发展社会事业，绥中、连山等县区通过国家义务教育均衡发展验收，承办国际花式台球锦标赛、中国铁人三项赛等重大赛事。推进重点工程建设，完成11所农村敬老院改造，市特教学校教学楼交付使用，30项重点民生工程基本完成。实施精准扶贫、精准脱贫，4.9万人、60个贫困村实现脱贫，建昌、兴城脱贫经验被全国推广。

　　(九)结合中央环保督察、国家海洋督察，深入推进生态环境治理。拆除燃煤小锅炉624台，淘汰黄标车5706辆，超额完成省定任务。实施首部地方水源保护条例，全面推进河长制，修复滨海生态系统，近岸海域水质达标率100%。完成人工造林25万亩、封山育林9万亩，治理水土流失面积10万亩。改善农村生态环境，省级生态村、生态乡镇分别达到119个和19个。解决了一批历史遗留、百姓关注的焦点难点问题，城乡生态环境明显改善。

　　(十)坚持依法行政，市县两级全面实施政府法律顾问制度。加强营商环境建设，在全省率先成立营商局，引入第三方评估，严肃查处典型案件，全市营商环境明显改善，得到省政府充分肯定。有力防控各类风险隐患，社会大局保持和谐稳定。有效遏制重特大生产安全事故，安全生产形势平稳向好。圆满完成十九大安保维稳任务，夺得全国综治最高奖“长安杯”。

　　坚持市委统一领导，严守制度规矩，落实各项决策部署，基本完成省委巡视反馈问题整改。主动接受人大依法监督、政协民主监督，代表建议、政协提案办理满意率99%以上。认真落实党中央和省委、市委全面从严治党决策部署，加强政府党的建设，严格落实“一岗双责”，推进“两学一做”学习教育常态化制度化，严厉查处违反中央八项规定精神和“四风”问题，转变工作作风，提高行政效能，政府系统担当意识、执政能力明显增强。

　　各位代表，成绩来之不易，凝聚着全市人民的智慧和汗水。在此，我代表市政府，向全市人民、人大代表、政协委员，向各民主党派、工商联、无党派人士、人民团体，向驻葫部队指战员、武警官兵、中省直单位和各界朋友，致以崇高的敬意和衷心的感谢!

　　回顾四年工作，我们也清醒地认识到，我市振兴发展还存在一些突出矛盾和问题。一是经济发展不平衡、不充分，经济总量小，外向度低。二是体制机制弊端没有根本解决，新旧动能转换速度慢。三是民生保障压力较大，民生方面短板较多。四是各类风险隐患依然存在，不容忽视。五是作风建设仍待加强，营商环境仍需改善。对此，我们将坚持问题导向，采取有效措施，全力加以解决。

　　三、今后五年工作目标和重点任务

　　各位代表!今后五年，是全面建成小康社会的决胜期，是实现“两个一百年”奋斗目标的历史交汇期。新时代、新起点，新使命、新征程，重任在肩、时不我待。我们将以习近平新时代中国特色社会主义思想为指导，深入贯彻落实党的十九大精神和习近平总书记系列重要讲话精神，坚持以人民为中心，坚持稳中求进工作总基调，按照高质量发展的要求，统筹推进“五位一体”总体布局，协调推进“四个全面”战略布局，认真落实新发展理念和“四个着力”“三个推进”要求，以供给侧结构性改革为主线，大力实施改革引领、创新驱动、结构优化、文化兴市、生态立市五大战略，统筹推进稳增长、促改革、调结构、惠民生、防风险各项工作，夺取全面建成小康社会的伟大胜利，全力开创生态宜居美丽富庶葫芦岛建设新局面!

　　今后五年政府工作的总体目标是：到2022年，经济总量突破千亿，力争达到1200亿元，居民收入不断增长，经济质量更加优化，城乡发展更加和谐，生态环境更加美好，人民生活更加幸福，城市综合实力进入全省第二阵营。

　　实现上述目标，需要我们加倍努力、务实工作，完成好五项重大任务。

　　(一)坚持协调发展，不断提高发展质量

　　落实“一带五基地”和“五大区域”发展战略，发挥比较优势，促进经济社会协调发展，推进城乡区域协调发展，加快产业协调发展，打造辽宁特色发展的新高地。

　　——深入推进供给侧结构性改革，促进三次产业融合发展，建立现代化经济体系。发展现代农业，完善流通体系，为经济发展提供稳定基础。拓展产业门类，改造传统产业，延长产业链条，推进军民融合，争做全省军民融合发展的先行者，打造装备制造、金属冶炼、精细化工、泳装服饰、新能源、新材料六大产业基地，为经济发展提供重要支撑。优化传统服务业，发展现代服务业，培育新兴业态，壮大旅游产业，为经济发展打造强劲引擎。

　　——实施乡村振兴战略，发展农村经济，壮大县域经济，建设产业兴旺、生态宜居、乡风文明、治理有效、生活富裕的社会主义新农村。优化城市布局，拓展城市空间，夯实城市承载，推进同城同质，实现三城同盛，让城乡更加富庶文明、和谐美丽。

　　——坚持用中国梦和社会主义核心价值观凝聚共识，加强思想道德建设，弘扬民族精神和时代精神，营造向上向善、孝老爱亲、诚信互助的社会风尚。深化文化体制改革，推进文化事业，繁荣文化产业，培育新型文化业态，为振兴发展汇聚强大精神力量。

　　(二)坚持创新发展，不断增强发展动力

　　实施创新驱动战略，完善体制机制，加快动能转换，优化创新资源，激活创新主体，以创新引领发展，建设创新型城市。

　　——坚决冲破思想束缚，清除体制机制障碍，加快体制机制创新，深入推进科技体制、国资国企、事业单位、审批制度、财税体制、教育卫生等重点领域改革，为经济社会发展提供强劲动力。

　　——深入实施中国制造2025葫芦岛行动计划，强化企业创新主体地位，加强知识产权保护利用，培育国家级科研中心。完善激励扶持政策，加快创新平台建设，引进培养各类人才和创新团队，释放全社会创新活力。

　　——创新政府管理，优化行政服务，提高工作效能，改善营商环境，营造创新发展的良好氛围。创新社会治理，健全制度体系，完善治理体制，构建共建共治共享的社会治理格局。

　　(三)坚持开放发展，不断优化发展结构

　　坚持以“一带一路”建设为统领，发挥区位优势，优化开放布局，面向两个市场，加强招商引资，推进区域合作，融入京津冀协同发展，建设全省对接京津冀的桥头堡、服务“一带一路”的示范区。

　　——承接全省港口资源整合，推进港口分工协作发展。加快港口建设，实现绥中港对外开放，融入全国口岸通关一体化。大力发展临港产业，建设港前物流园区，拉动腹地城区发展，逐步实现“港产城”一体化。

　　——完善开放载体，加快沿海经济带开发开放，培育主导产业，发展产业集群，创新管理机制，打造特色园区，实现园区经济总量翻两番，成为经济发展的重要增长极。

　　——复制推广自贸区经验，探索建设综合保税区。培育新兴出口产业集群，打造出口知名品牌。推动国际产能合作，积极开拓国际市场，扩大外贸进出口，提高经济外向度，打造葫芦岛竞争新优势。

　　(四)坚持绿色发展，不断建设美丽家园

　　加快生态文明建设，坚定走生产发展、生活富裕、生态良好之路，把蓝天、绿水、青山、净土还给人民群众，留给子孙后代。

　　——实行最严格的生态环境保护制度，决不逾越生态保护红线。大力发展节能环保、清洁能源、循环产业，积极倡导简约适度、绿色低碳生活方式，实现城市永续发展。

　　——着力解决环境问题，突出大气污染防治，保护整治海岸线和河流生态环境，加强土壤污染治理，坚决打赢污染防治攻坚战。

　　(五)坚持共享发展，不断创造美好生活

　　抓住人民最关心最直接最现实的利益问题，既尽力而为，又量力而行，一件事情接着一件事情办，一年接着一年干，不断满足人民群众对美好生活的向往。

　　——坚持就业优先、鼓励创业，健全多层次社会保障体系。优先发展教育，推动城乡义务教育一体化。建设健康葫芦岛，为百姓提供全方位全生命周期健康服务。完善社会治安防控体系，建设平安葫芦岛。

　　——加强社区基础设施建设，完善社区治理体系，加强社区党建工作，健全社区服务功能，促进社区基本服务均等化、优质化，满足居民多样化服务需求，使社区成为社会治理的坚实平台。

　　——坚持“六个精准”“五个一批”，确保6.03万贫困人口、82个省级贫困村如期脱贫，坚决打赢脱贫攻坚战，与全国同步迈进小康社会。

　　各位代表!我们相信，在市委的坚强领导下，在市人大市政协的监督支持下，在全市人民的共同努力下，我们的奋斗目标一定能够实现!

　　四、2018年工作安排

　　今年是贯彻党的十九大精神的开局之年，也是决胜全面建成小康社会的关键之年，做好今年工作，意义重大。我们的工作目标是地区生产总值增长7%;规模以上工业增加值增长8%;固定资产投资增长13.5%;一般公共预算收入增长10%;社会消费品零售总额增长6%;城镇和农村居民人均可支配收入均增长6.5%。

　　我们将全力做好十一项重点工作：

　　(一)狠抓项目建设，拉动有效投资

　　继续开展“项目年”活动，落实包扶机制，完善激励制度，加强要素保障，确保重点项目顺利实施。安排亿元以上重点项目200个，总投资2100亿元，完成投资210亿元。徐大堡核电一期、锌业股份20万吨电解锌等70个项目开工建设，大唐热电联产、北方铜业电解铜等60个项目竣工投产。

　　狠抓招商引资，盯住京津冀抢转移项目，对接江苏、长三角、珠三角要合作项目，咬住外商外资引进外资项目，关注中央政策申报项目，全年引进内资160亿元，实际利用外资1亿美元。

　　(二)狠抓实体经济，提升工业经济实力

　　深化工业供给侧结构性改革，拓展产业门类，挖掘经济增长点。实施工业重点项目54个，30万吨燃料乙醇、锦西石化醚化技改等10个项目建成投产。发展新兴产业，加快建设明盛数码彩晶玻璃、泰德尔楼宇自控等5个重点项目。延长产业链条，实施中冶葫芦岛有色30万吨铜冶炼、孚迪斯高温复合材料等22个项目。推进军民融合，开工建设航空有机玻璃等重点项目13个，军民融合产业示范园初步形成规模。放大泳装产业优势，建设泳装研发、生产和销售基地，创建国家知名品牌示范区，努力成为标准制定者和行业风向标。

　　(三)狠抓第三产业，加快现代服务业发展

　　推进传统服务业提质升级、产业集聚、转型发展。优化传统产业，加快兴隆大家庭等商贸综合体建设，新增规上服务业企业20家、限上商贸企业50家。发展新兴业态，重点培育金融、会展、新型地产、健康养老、社区服务、文化创意、体育产业等新模式新业态，加快推进二手车等各类交易市场建设。推进园区发展，建设东城商贸物流集聚区、兴城滨海服务业新业态集聚区。壮大电商产业，创建国家电子商务示范市。

　　继续实施“六个一工程”，建设丽汤首山温泉、亲海玻璃栈道等重点旅游项目，改造提升九门口长城、兴城古城等景区。保护传统古村落，发展乡村旅游。完善基础设施，改造锥子山长城等景区道路。提高服务质量，发展智慧旅游。改善旅游环境，实施东戴河景区综合整治。旅游总收入增长25%，对接京津冀旅游先行区取得实质性进展。

　　(四)狠抓农村经济，推进乡村振兴进程

　　继续落实“五个围绕”，壮大农村集体经济，推进农业产业化发展。调减玉米面积，发展设施农业、认养农业、生态农业。打造品牌果业，发展精品果园。发展现代化养殖，推进北京大北农畜牧、新加坡林虾集团智能水产养殖等项目。发展农产品深加工产业，加快建设35万吨大豆精深加工、大宇调味品扩建等农业产业化项目。推进产业集聚发展，加快兴城优质花生出口示范区、建昌食品产业园建设，兴城农产品加工集聚区达到省级标准。推进特色乡镇试点，打造一批产业鲜明、环境优美的特色乡村。

　　(五)狠抓各项改革，破除体制机制障碍

　　推进国资国企改革，加快企业集团混合所有制改造。深化“放管服”改革，推进东戴河项目审批承诺制试点。深化行政体制改革，优化设置，压缩编制。推进市县两级事业单位改革。推进农村综合改革，完善承包地三权分置制度，完成土地确权登记。推进县乡财税体制改革，加大对县乡财政转移支付补助。完成省定“三去一降一补”任务。

　　(六)狠抓对外开放，提高经济外向度

　　把沿海经济带作为对外开放主战场，贯彻落实沿海经济带三年攻坚计划，培育主导产业，发展特色园区。明晰产业定位，东戴河新区重点发展信息技术、生物工程和临港产业;北港工业区重点发展精细化工、装备制造产业;滨海经济区重点发展高新技术产业、泳装全产业链和农产品精深加工;打渔山园区重点发展高端流体设备、精密仪器产业;海洋工程工业园重点发展军民融合产业。实施项目支撑，加快建设毕氏集团硅锰合金、思科赛斯高铁配件等重点项目。推进园区体制机制改革，实行扁平化管理、公司化运营。

　　主动融入“一带一路”建设，推进与招商局集团合作，加快港口建设。葫芦岛港开工建设疏港铁路专用线，大力发展临港产业，加快推进300万吨大豆深加工、低温丙烷等重点项目;绥中港加快建设港前物流园区、产业园区，实现对外开放。扩大利用外资，促进进出口贸易，鼓励对外投资，构建对外开放新格局。

　　(七)狠抓县域经济，筑牢县区发展根基

　　落实县域经济发展三年行动计划，整合优势资源，制定产业规划，明确功能定位，走城乡统筹、产城融合、特色发展之路，发挥辐射作用，带动乡村振兴。县(市)区发展园区经济，重点实施牵动型项目。乡镇突出本地优势，重点实施特色项目。村屯壮大集体经济，重点实施强村富民项目。把发展县域经济的着力点放在民营经济上，消除体制障碍，突出要素保障，健全政策体系，搭建服务平台，引导金融资源流向民营经济，提振民营经济发展信心，做大民营经济总量。

　　(八)狠抓城市建设，提升城市发展水平

　　继续实施三城同盛，改善生态环境，改造城中村，治理城中河，完善城市路网。实施“暖房子”工程，持续改造棚户区、老旧小区。整治连山河、茨山河、五里河城区段，建设三河入海口生态湖。规划建设夹山隧道，加快建设高铁高桥站，建成兴城西客站。完成外环路提升工程，建成海韵路。延伸文兴路、高新五路，改造茨齐路，打通滨河路，畅通城市道路，提升城市品质，彰显城市魅力。

　　继续提升城市承载。加快基础设施建设，完善城市功能，增加土地储备。扩建老区污水处理厂，实施龙湾大街雨污分流改造，开工建设垃圾发电厂。完善消火栓、供水喉等消防基础设施，建设消防训练基地。

　　加强城市精细化管理。完善城市管理体制，细化城市管理内容，提高城市管理能力，建设智慧城市。推进城区市场建设，治理马路市场。开通外环路公交，实现城区出租车互通，建设便民停车设施。推进城市管理网格化，将城市管理向社区延伸，让城市更加方便、清洁、有序、文明。

　　(九)狠抓污染防治，改善城乡人居环境

　　深入整改中央环保督察反馈问题，大力推进“青山碧水蓝天净土”工程。启动“智慧环保”工程，建成大气监管预警平台。全面淘汰城区10吨以下燃煤小锅炉，继续淘汰黄标车和老旧车辆，加强秸秆禁烧管控，环境空气质量达标率达到72.6%。全面落实河长制，全市河流水质优良比例达到50%以上，近岸海域水质状况保持稳定。编制海岸线保护规划，实施滨海生态环境治理工程。完成青山水库环保工程，修复稻池村和八家子污染土壤，整治青山水库、女儿河上游生态环境，建设标准化自然保护区。整治农村人居环境，防治农业面源污染，坚决打好污染防治攻坚战。

　　(十)狠抓民生改善，提高百姓幸福指数

　　健全社保体系。继续提高城乡低保标准，确保养老金按时足额发放，全面实施跨省住院费用联网直接结算。促进就业创业，城镇登记失业率控制在5%以内。发展住房租赁市场，降低商品房库存。完善便民服务设施，加快市民服务中心建设。突出抓好社区治理，加强标准化建设，开展示范社区创建活动，提升社区服务能力和水平。

　　发展社会事业。实施“智慧教育”工程，建昌、南票通过国家义务教育均衡发展验收。抓好婴幼儿照护和儿童早期教育，解决学生课外负担重、“择校热”“大班额”问题。深化医疗卫生体制改革，推进分级诊疗，建设标准化村卫生室。建设基层文化服务中心，丰富城乡群众文化生活。落实人才政策，设立人才引进基金，打造葫芦岛新型智库。

　　加快整体脱贫。坚持精准扶贫、精准脱贫，实施“五个联动”，落实“五个一批”，推进产业扶贫、金融扶贫、技能脱贫，强化驻村帮扶，形成脱贫攻坚合力，确保3.7万人、65个贫困村脱贫。

　　强化风险防范。健全风险防范预警机制，落实各类管控措施，明确监管责任，严肃督查问责，确保不发生养老金支付、政府性债务、企业金融债务等各类风险，坚决打好防范化解重大风险攻坚战。

　　加强社会治理。落实安全生产三个责任，坚决遏制重特大生产安全事故发生。落实信访主体责任，实现“减存控增”。继续创建全国双拥模范城市、全国文明城市、国家卫生城市、国家食品安全示范城市。

　　坚持民生为本。以改善环境、治理河流为重点，以便民、利民、惠民为目标，将财政支出的80%以上用于民生建设，扎实做好十大民生工程、28件民生实事，确保中央各项惠民政策彻底贯彻落实。

　　(十一)狠抓自身建设，提高政府执行力

　　树立法治思维，坚持依法行政，严格遵循重大行政决策法定程序。强化服务意识，实施营商环境建设计划，持续开展营商环境建设年活动，打造“营商环境关外第一市”。弘扬实干精神，激发创业激情，让苦干、实干成为政府干部的鲜明特质。增强“八个本领”，狠抓工作落实，做到主动抓落实、敢于抓落实、善于抓落实、坚决抓好落实，保证各项决策部署落到实处。

　　坚决服从市委领导，自觉接受人大依法监督、政协民主监督，主动接受社会监督。加强政府党的建设，坚持“一岗双责”，严格履行全面从严治党主体责任，严格落实意识形态工作责任制。加强党风廉政建设，严格落实中央八项规定精神，严防“四风”反弹回潮，切实防止“十种新表现”，保护风清气正的政治生态。

　　各位代表，进入新时代，踏上新征程，把握新机遇，谋求新发展，这是我们的历史使命，更是神圣职责。让我们更加紧密地团结在以习近平同志为核心的党中央周围，不忘初心、牢记使命，锐意进取、埋头苦干，为全面建成小康社会、建设生态宜居美丽富庶葫芦岛而努力奋斗!
